# MicroRNA-148a-3p in pericyte-derived extracellular vesicles improves erectile function in diabetic mice by promoting cavernous neurovascular regeneration

**DOI:** 10.1186/s12894-023-01378-4

**Published:** 2023-12-16

**Authors:** Jiyeon Ock, Fang-Yuan Liu, Fitri Rahma Fridayana, Lashkari Niloofar, Minh Nhat Vo, Yan Huang, Shuguang Piao, Tie Zhou, Yin Guonan

**Affiliations:** 1 Department of Urology and National Research Center for Sexual Medicine, Inha University School of Medicine, 7-206, 3rd ST, Shinheung-Dong, Jung-Gu, Incheon, 22332 Republic of Korea; 2 Program in Biomedical Science & Engineering, Inha University, Incheon, South Korea; 3 Department of Urology, Changhai Hospital Affiliated with the Naval Medicine University, Shanghai, 200433 People’s Republic of China; 4 Department of Urology, Shanghai Fourth People’s Hospital, School of Medicine, Tongji University, No. 1279 Sanmen Road, Shanghai, 200434 China

**Keywords:** Diabetes, Erectile dysfunction, MCPs-EVs, miR-148a-3p, Neurovascular regeneration, PDK4

## Abstract

**Background:**

To investigate the regulatory role of microRNA (miR)-148a-3p in mouse corpus cavernous pericyte (MCPs)-derived extracellular vesicles (EVs) in the treatment of diabetes-induced erectile dysfunction (ED).

**Methods:**

Mouse corpus cavernous tissue was used for MCP primary culture and EV isolation. Small-RNA sequencing analysis was performed to assess the type and content of miRs in MCPs-EVs. Four groups of mice were used: control nondiabetic mice and streptozotocin-induced diabetic mice receiving two intracavernous injections (days − 3 and 0) of phosphate buffered saline, MCPs-EVs transfected with reagent control, or MCPs-EVs transfected with a miR-148a-3p inhibitor. miR-148a-3p function in MCPs-EVs was evaluated by tube-formation assay, migration assay, TUNEL assay, intracavernous pressure, immunofluorescence staining, and Western blotting.

**Results:**

We extracted EVs from MCPs, and small-RNA sequencing analysis showed miR-148a-3p enrichment in MCPs-EVs. Exogenous MCPs-EV administration effectively promoted mouse cavernous endothelial cell (MCECs) tube formation, migration, and proliferation, and reduced MCECs apoptosis under high-glucose conditions. These effects were significantly attenuated in miR-148a-3p-depleted MCPs-EVs, which were extracted after inhibiting miR-148a-3p expression in MCPs. Repetitive intracavernous injections of MCPs-EVs improved erectile function by inducing cavernous neurovascular regeneration in diabetic mice. Using online bioinformatics databases and luciferase report assays, we predicted that pyruvate dehydrogenase kinase-4 (PDK4) is a potential target gene of miR-148a-3p.

**Conclusions:**

Our findings provide new and reliable evidence that miR-148a-3p in MCPs-EVs significantly enhances cavernous neurovascular regeneration by inhibiting PDK4 expression in diabetic mice.

**Supplementary Information:**

The online version contains supplementary material available at 10.1186/s12894-023-01378-4.

## Background

Erectile dysfunction (ED) is a vascular disease, which is estimated to affect 322 million men worldwide by 2025, and 30–75% of men experience ED, especially diabetes-related ED [1]. Oral phosphodiesterase 5 (PDE5) inhibitors, which rely on endogenous nitric oxide (NO) bioavailability, constitute the first-line treatment for diabetes-induced ED [2]. However, long-term microangiopathy in erectile tissues of patients with diabetes mellitus (DM) can induce hypoxia and structural changes, which cause endothelial-pericyte dysfunction and peripheral neuropathy [3] and may lead to poor responses to oral PDE5 inhibitors [4]. The clinical development of several novel targets, including COMP-Ang1 [5], vascular endothelial growth factor [6], and brain-derived neurotrophic factor [7], have been hindered due to insufficient efficacy, side effects, and protein engineering difficulties in preclinical studies. Therefore, an efficient therapeutic strategy with minimal adverse effects is needed in the complicated pathology of angiopathy and neuropathy in diabetes-induced ED.

Extracellular vesicles (EVs) are released by almost all cell types [8] and comprise proteins, lipids, messenger RNAs (mRNAs), microRNAs (miRNAs), and nucleic acids, which play multiple roles in the physiological and pathological communication between donor and recipient cells [9]. EV-mimetic nanovesicles (NV) isolated from mouse embryonic stem cells and mouse corpus cavernous pericytes (MCPs) significantly induce neurovascular regeneration in diabetes-induced and cavernous nerve injury (CNI)-induced ED mouse models [10, 11]. However, the detailed pathways and target genes regulated by MCP*-*derived EVs (MCPs-EVs) remain unelucidated. Moreover, miRNAs repress or degrade mRNA at the post-transcriptional level, thereby regulating various cellular processes, such as angiogenesis, proliferation, apoptosis, and neural regeneration [12, 13]. We hypothesized that MCPs-EVs promote neurovascular regeneration by delivering miRNAs, thereby improving erectile function in diabetic mice.

This study primary objectives were conducted to determine the type and content of miRNAs in MCPs-EVs as well as the neurovascular regenerative function under diabetic conditions; and secondary objectives was to determine the target genes of miRNAs in MCPs-EVs.

## Methods

### Ethics statement and study design

This animal model study included 75 adult male C57BL/6 J mice (8 weeks old; Orient Bio, Inc.): 15 for the MCPs primary culture, MCPs-EVs isolation, and small-RNA sequencing analysis; 20 for in vitro studies; and 40 for in vivo studies. All animal experiments were approved by the Ethics Committee at the Inha University (approval number: INHA 200309-691). Animals were monitored daily for health and behavior as previously described [14, 15]. In all studies, animals were anesthetized with an intramuscular injection of ketamine (100 mg/kg; Yuhan Corp., Seoul, Korea) and xylazine (5 mg/kg; Bayer Korea, Seoul, Korea). Animals were euthanized by 100% CO_2_ gas replacement; cessation of heartbeat and respiration were confirmed prior to harvesting tissue. All animal studies were randomized and blinded. No mice died during the experimental procedures.

### Primary culture of MCPs and MCECs and treatment

Primary MCPs and MCECs were prepared from mouse penile tissue as previously described [16, 17]. Briefly, 8 weeks old male C57BL/6 J mice were euthanized by 100% CO_2_ gas replacement. Then, the penis tissues were harvested and maintained in sterile vials with HBSS (Gibco). After washing with PBS for 3 times, the urethra and dorsal neurovascular bundle were removed, and only the corpus cavernosum tissues were used. For MCPs culture, the corpus cavernosum tissues were cut into approximately 1-2 mm sections and settled via gravity into collagen I-coated 35 mm cell culture dishes with 300 μL complement DMEM (GIBCO) at 37 °C for 20 min in a 5% CO_2_ atmosphere. Then, 900 μL of complement medium was added and incubated at 37 °C with 5% CO_2._ The complement medium contained 20% FBS, 1% penicillin/streptomycin, and 10 nM human pigment epithelium-derived factor (PEDF; Sigma-Aldrich). The medium was changed every 2 days, and after approximately 10 days sprouting cells were sub-cultured into collagen I (Advanced BioMatrix, San Diego, CA, USA)-coated dishes. For MCECs culture, corpus cavernosum tissues were cut into approximately 1-2 mm sections, put into 60 mm dishes, and covered by Matrigel (Becton Dickinson, Mountain View, CA, USA). Tissues were cultured with the complement M199 medium (Gibco) containing 20% fetal bovine serum (FBS, Gibco), 0.5 mg/mL of heparin (Sigma-Aldrich), 5 ng/mL of recombinant human vascular endothelial growth factor (VEGF, R&D Systems Inc., Minneapolis, MN, USA), and 1% penicillin/streptomycin (Gibco) in a 5% CO2 atmosphere incubator at 37 °C. After cells were confluent on the bottom of the 60 mm cell culture dishes (approximately 2 weeks of culture), sprouting cells were sub-cultured into other cell culture dishes coated with 0.2% gelatin (Sigma-Aldrich, St Louis, MO, USA). Cells from passages 2 to 3 were used for the experiments. Diabetes-induced angiopathy was mimicked by serum-starving cells (MCECs and MCPs) for 24 hours and then exposing them to normal-glucose (NG; 5 mM glucose, Sigma-Aldrich, St. Louis, MO, USA) or high-glucose (HG; 30 mM glucose) conditions for 3 days [18].

### MCPs-EV isolation, quantitation, and identification

MCPs-EVs were isolated from DMEM-cultured MCPs (Gibco; Carlsbad, CA, USA) in medium supplemented with 20% exosome-depleted FBS (Gibco), with 1% penicillin/streptomycin (Gibco), using a commercial EV isolation kit (ExoQuick-TC, System Biosciences, LLC., Palo Alto, CA, USA) according to the manufacturers’ instructions. The EXOCET exosome quantitation kit (System Biosciences, LLC.) was used to quantify MCPs-EVs, and their concentration was adjusted to 1 μg/μL before treatments.

The MCPs-EV morphology was ascertained by transmission electron microscopy (TEM; Electron Microscopy Sciences, Fort Washington, PA, USA) and their characterization was validated based on the expression of one negative and three positive EV markers in Western blotting [11] (negative marker: GM130 [1:1000; BD Biosciences, San Jose, CA, USA]; Three positive EV markers [1:1000]: CD9 [Abcam, Cambridge, MA, USA), CD63 [NOVUS Biologicals, Littleton, CO, USA], and CD81 [NOVUS Biologicals]).

### Fluorescence dye labeling of the MCPs-EVs for tracking analysis

MCPs-EVs were labelled with 1,1′-dioctadecyl-3,3,3′,3′-tetramethylindodicarbocyanine, 4-chlorobenzenesulfonate salt (DiD) red-fluorescent dye (ThermoFisher Scientific, Inc., Carlsbad, CA, USA) according to the manufacturer’s instructions. After 6-hour DiD-labeled MCPs-EV treatment, MCECs were fixed by incubation with 4% formaldehyde for 15 minutes. DiD dye tracking was determined under a confocal fluorescence microscope (K1-Fluo, Nanoscope Systems, Inc., Daejeon, Korea).

### miRNA identification by small-RNA sequencing analysis

The small-RNA-sequencing assay was performed by E-Biogen Inc. (Korea). For control and test RNAs, a library was constructed using the NEBNext Multiplex Small RNA Library Prep kit (New England BioLabs, Inc., USA) according to the manufacturer’ instructions. The small-RNA-sequencing data were deposited in the Gene Expression Omnibus database (www.ncbi.nlm.nih.gov/geo; accession no. GSE195533).

### Transfection of MCPs with miR148a-3p inhibitors

Primary MCPs were transfected with 20 nM miR148a-3p inhibitor (a mirVana® miRNA inhibitor, Cat #, 4,464,084, Thermo Fisher, San Jose, CA, USA) using Lipofectamine 2000 (Invitrogen, Carlsbad, CA, USA). After 24 hours, the culture medium was replaced with DMEM supplemented with 20% exosome-depleted FBS with 1% penicillin/streptomycin for 3 days, and the culture medium was collected for conditioned MCPs-EV isolation.

### Extraction of RNA in MCPs-EVs

Exosomal RNA was isolated from 150 ul of MCPs-EVs using the miRNeasy Serum/Plasma Kit (Qiagen, Hilden, Germany) according to the manufacturer’s protocol as described previously [19].

### Real-time PCR (qPCR)

Total RNA from MCPs was isolated using TRIzol (Invitrogen) according to the manufacturer’s instructions. miRNAs were reverse-transcribed using the Taqman microRNA Reverse Transcription kit (Applied Biosystems, Carlsbad, CA, USA) and the associated miRNA-specific stem-loop primers (Applied Biosystems). The RT reaction conditions were: 30 minutes at 16 °C to anneal primers, 30 minutes at 42 °C for extension of primers on miRNA and synthesis of the first cDNA strands, and 5 minutes at 85 °C to stop the reaction. qPCR was performed on the 7500 Fast Real-Time PCR System (Applied Biosystems) with the following conditions: one denaturing step at 95 °C for 10 minutes, followed by 40 denaturing cycles at 95 °C for 15 seconds; and annealing and elongation at 60 °C for 60 seconds. The data presented correspond to the mean of 2^-ddct^ from at least three independent experiments after normalization to U6.

### Measurement of nitric oxide (NO) levels

The nitrite assay kit (MAK367, Sigma-Aldrich) was used to determine nitric oxide (NO) concentration in MCECs, according to the manufacturer’s protocol as described previously [14]. MCECs were seeded in 6-well plates at a density of 5 × 10^5^ cells/well in 2 mL of M199 medium. After 24 hours, MCECs were exposed to glucose conditions with or without MCPs-EV or miR-148a-3p-depleted MCPs-EV for 72 hours at 37 °C in a humidified 5% CO_2_ atmosphere. Then, cultured medium was collected for NO concentration measurement. Nitrite levels was measured at a wavelength of 540 nm, using a microplate spectrophotometer (BioTek Instruments Inc., Winooski, VT, USA). Each experiment contained six replicates and repeated four times.

### Tube-formation assay

Tube-formation assays were performed as described previously [20]. Tube formation was monitored for 18 hours under a phase-contrast microscope (CKX41, Olympus, Tokyo, Japan). The number of master junctions from four separate experiments were quantified using Image J (National Institutes of Health [NIH] 1.34, http://rsbweb.nih.gov/ij/).

### Cell-migration assay

The migration assay was performed with the SPLScar™Block system (SPL life sciences, Pocheon-si, Gyeonggi-do, Korea) on 60-mm culture dishes [20]. Images were obtained using a phase-contrast microscope (Olympus), and cell migration was analyzed by determining the ratio of cells that moved into the frame line in the figures from four separate block systems using Image J.

### TUNEL assay

The terminal deoxynucleotidyl transferase-mediated deoxyuridine triphosphate nick-end labeling (TUNEL) assay was performed using the ApopTag® Fluorescein In Situ Apoptosis Detection Kit (Chemicon, Temecula, CA, USA) as described previously [21]. Samples were mounted in a solution (Vector Laboratories Inc., Burlingame, CA, USA) containing 4,6-diamidino-2-phenylindole (DAPI), a nuclear stain. Digital images were obtained using a confocal fluorescence microscope (K1-Fluo; Nanoscope Systems, Inc). The number of apoptotic cells was counted using Image J.

### Animal treatment and measurement of erectile function

Diabetes was induced in 8-week-old C57BL/6 J mice by injecting them with streptozotocin (STZ; 50 mg/kg, Sigma-Aldrich) for 5 consecutive days [22]. After 8 weeks, mice were randomly distributed into four groups: control nondiabetic mice and STZ-induced diabetic mice receiving two successive intracavernous injections of PBS (20 μL; days − 3 and 0), two successive intracavernous injections of MCPs-EV control reagent (5 μg in 20 μL PBS, days − 3 and 0), or two successive intracavernous injection of miR-148a-3p-depleted MCPs-EV (5 μg in 20 μL PBS, days − 3 and 0) into the midportion of the corpus cavernosum (*n* = 5 per group). For injections, mice were anesthetized with intramuscular injections of ketamine (100 mg/kg) and xylazine (5 mg/kg) and positioned supine on a thermoregulated surgical table and the base of the penis was compressed with a vascular clamp before injection. After injection, the clamp was left in place for 30 minutes to prevent backflow of blood from the penis.

Two weeks later, the erectile function measurement was performed as previously described [22]. Systemic blood pressure was measured continuously by using a noninvasive tail-cuff system (Visitech Systems, Apex, NC, USA) before the measurement of intracavernous pressure (ICP). The ratios of maximal ICP and total ICP to mean systolic blood pressure (MSBP) were calculated to normalize for variations in systemic blood pressure.

### Histological examination

For fluorescence microscopy, mice penile tissue was fixed in 4% paraformaldehyde for 24 hours at 4 °C, and the frozen tissue sections (12-μm thick) were incubated overnight at 4 °C with primary antibodies including: CD31 (endothelial cell marker, 1:50; Millipore, Temecula, CA, USA), NG2 antibody (pericyte marker, 1:50; Millipore), β (III)-tubulin antibody (neuronal cell marker, 1:200; Abcam), nNOS (neuronal cell marker, 1:100; Santa Cruz Biotechnology Inc., Dallas, TX USA), or phospho-Histone H3 (PH3; Mitosis marker, Upstate Biotechnology Inc., Temecula, CA, USA). After several washes with PBS, samples were incubated with donkey anti-rabbit DyLight® 550 (1:200; Abcam), goat anti-Armenian hamster Fluorescein isothiocyanate (FITC; 1:200; Jackson ImmunoResearch Laboratories, West grove, PA, USA), donkey anti-rabbit FITC (1:200; Jackson ImmunoResearch Laboratories), and donkey anti-chicken Tetramethylrhodamine (TRITC) secondary antibodies (1:200; Jackson ImmunoResearch Laboratories) for 2 hours at room temperature. Using a DAPI-based solution (Vector Laboratories Inc.), samples were mounted for nuclear staining. Samples were visualized and images were obtained with a confocal microscope (Nanoscope Systems, Inc). Quantitative analysis was performed using Image J.

### Target prediction with bioinformatics

Computational predictions of miRNA target genes were obtained using the following published algorithms: DIANA-microT-CDS (http://www.microrna.gr/microT-CDS), TargetScan (http://www.targetscan.org), and miRDB (http://www.mirdb.org).

### Luciferase miRNA target reporting assays

A PDK4 3’UTR target plasmid (50 ng, GeneCopoeia, Rockville, MD, USA) or a negative control vector (50 ng, GeneCopoeia) with miR148a-3p mimic (50 nM, Ambion) or a control mimic (50 nM, Ambion) were co-transfected into MCECs using Lipofectamine2000 transfection reagent. After 48 hours, the cells were lysed and luciferase activities were measured using the Dual-Luciferase Reporter Assay Kit 2.0 (GeneCopoeia) and a luminometer (BioTek Instruments Inc., Winooski, VT, USA). Relative luciferase activity was calculated by normalizing the *Renilla* Luciferase signal against that of firefly luciferase.

### Western blotting analysis

Equal amounts of protein (30 μg per lane) were subjected to 4–20% SDS-PAGE and then transferred to PVDF membranes. After blocking with 5% non-fat dry milk for 1.5 hours at room temperature, the membranes were incubated at 4 °C overnight with the following primary antibodies: pyruvate dehydrogenase kinase-4 (*PDK4*; 1:1000; NOVUS Biologicals) and β-actin (1:4000; Santa Cruz Biotechnology). The membranes were washed thrice for 10 min each with PBST at room temperature. Subsequently, the membranes were incubated with goat anti-rabbit IgG H&L (HRP; 1:1000; Abcam), donkey anti-goat IgG H&L (HRP; 1:1000; Abcam), or goat anti-mouse IgG H&L (HRP; 1:1000; Abcam) secondary antibodies for 2 hours at room temperature, and signals were visualized using an ECL detection system (Amersham Pharmacia Biotech, Inc.). The results were quantified by densitometric analysis using Image J.

### Statistical analysis

All results are expressed as the mean ± SEM of at least four independent experiments. The unpaired *t*-test was used to compare two groups, and one-way ANOVA followed by Tukey’s post hoc test was used for four-group comparisons. The analysis was conducted using GraphPad Prism version 8 (Graph Pad Software, Inc.). *P* values less than 0.05 were considered statistically significant. Statistical sample sizes were determined based on our previous studies [15, 23]. In vivo functional evaluation requires at least 5 animals per group, and other experiments require at least 4 samples per group for more effective statistical analysis.

## Results

### MCPs-EV characterization and tracking analysis in MCECs

**Fig. 1:**
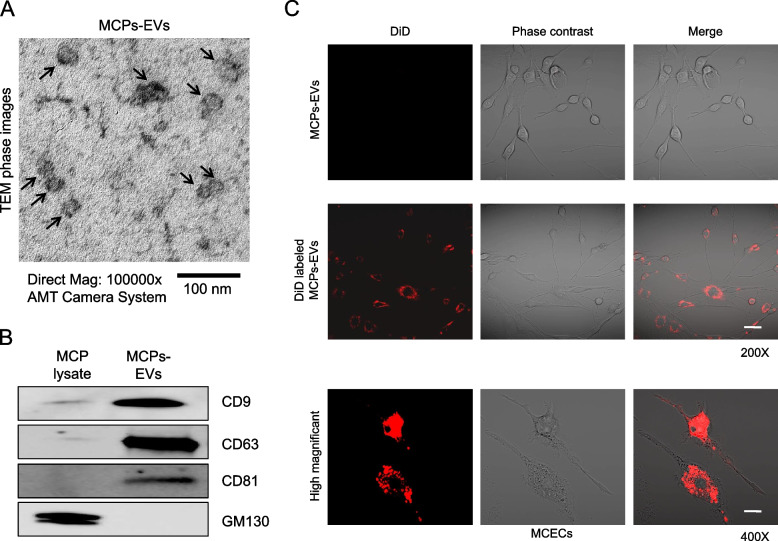
MCP-derived extracellular vesicle (MCPs-EV) characterization and tracking analysis in MCECs. **a** Representative transmission electron micrograph (TEM) phase images for detecting isolated MCPs-EVs as indicated by the arrows. Scale bar = 100 nm. **b** Representative Western blot for three positive EV markers (CD9, CD63, and CD81) and one negative EV marker (GM130) in MCP lysate and MCPs-EVs. **c** DiD-labeled MCPs-EVs (red) were treated with MCECs for 6 h. Scale bar = 50 μm. MCPs, mouse corpus cavernous pericyte; MCECs, mouse cavernous endothelial cells; DiD, 1,1′-dioctadecyl-3,3,3′,3′-tetramethylindodicarbocyanine, 4-chlorobenzenesulfonate salt

Based on the TEM images, the MCPs-EVs exhibited a unique cup-shaped morphology (diameter ~ 30 nm; Fig. 1a). Western blotting showed that positive EVs surface markers, such as CD9, CD63, and CD81, were present in MCPs-EVs but had low expression levels in MCP lysate. Conversely, negative EV surface markers, such as GM130, were not detected in purified MCPs-EVs (Fig. 1b). To determine whether the secreted MCPs-EVs were taken up by endothelial cells, DiD red dye-labeled MCPs-EVs were treated in MCECs and were detected inside the MCECs after 6 hours (Fig. 1c). These data suggest that MPCs-EVs are taken up by MCECs.

### MicroRNA profiling of MCPs-EVs

To assess the composition of miRNAs in MCPs-EVs, we performed small-RNA sequencing analysis with MCPs-EVs. We detected only 90 miRNAs in MCPs-EVs, and the top 10 expressed miRNAs are listed in Table 1. We focused on miR-148a-3p, the most abundant miRNA in MCPs-EVs.

**Table 1:** Small RNA sequencing analysis of Mouse cavernous pericytes (MCPs)-derived extracellular vesicles (EVs)

Gene symbol	MCPs-EVs Raw data (RC)	Sequence
**mmu-miR-148a-3p**	**15,453**	**UCAGUGCACUACAGAACUUUGU**
mmu-miR-7051-5p	13,658	UCACCAGGAGGAAGUUGGGUCA
mmu-miR-125a-5p	3847	UCCCUGAGACCCUUUAACCUGUGA
mmu-miR-151-3p	3773	CUAGACUGAGGCUCCUUGAGG
mmu-miR-5110	3136	GGAGGAGGUAGAGGGUGGUGGAAUU
mmu-miR-486b-5p	1559	UCCUGUACUGAGCUGCCCCGAG
mmu-miR-486a-5p	1364	UCCUGUACUGAGCUGCCCCGAG
mmu-miR-21a-5p	609	UAGCUUAUCAGACUGAUGUUGA
mmu-miR-191-5p	546	CAACGGAAUCCCAAAAGCAGCUG
mmu-miR-5126	372	GCGGGCGGGGCCGGGGGCGGGG

### Inhibition of miR-148a-3p expression reduces the angiogenic effect of MCPs-EVs under high-glucose conditions

**Fig. 2:**
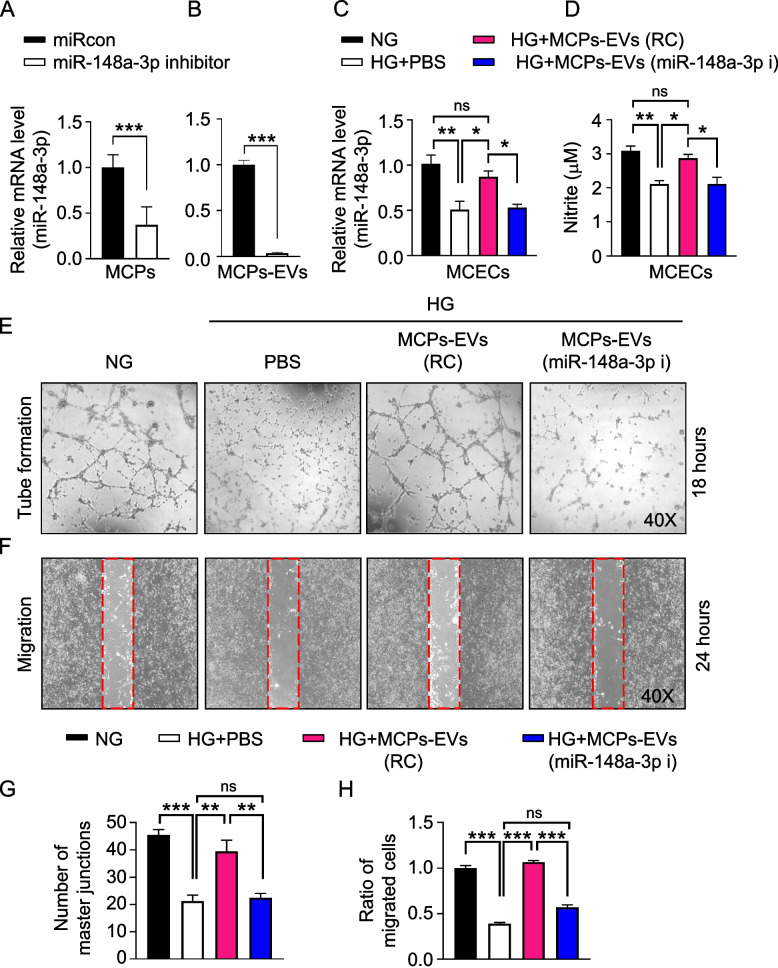
MCPs-EVs induces angiogenesis in MCECs through miR-148a-3p. **a** The mRNA levels of miR-148a-3p decreased in MCPs transfected with miR-148a-3p inhibitor compared to the microRNA control (miRcon). **b** The mRNA levels of miR-148a-3p decreased in MCPs-EVs isolated from conditioned MCPs after transfected with miR-148a-3p inhibitor compared to the microRNA control (miRcon). **c** and **d** The miR-148a-3p mRNA levels (**c**) and nitrite production (**d**) in MCECs treated with PBS, MCPs-EVs-regent control (MCPs-EVs-RC, 1 μg/mL), MCPs-EVs-miR-148a-3p inhibitor (MCPs-EVs-miR-148a-3p-i, 1 μg/mL) under normal-glucose (NG) and high-glucose (HG) conditions for 3 days. **e** Tube-formation assay was performed in MCECs treated with PBS, MCPs-EVs-regent control (MCPs-EVs-RC, 1 μg/mL), MCPs-EVs-miR-148a-3p inhibitor (MCPs-EVs-miR-148a-3p-i, 1 μg/mL) under normal-glucose (NG) and high-glucose (HG) conditions for 3 days; representative images obtained at 18 hours (screen magnification, 40×). **f** Migration assay was performed in MCECs with the same treatment conditions as for tube formation; representative images were obtained at 24 hours (screen magnification, 40×). **g** Number of master junctions were quantified using Image J and the results are presented as mean ± SEM (*n* = 4). **h** Ratio of cells that migrated into the red-dotted frame were quantified using Image J and the results are presented as mean ± SEM (*n* = 4). The value expressed as ratios of the NG group was set to 1. **p < 0.01; ***p < 0.001. MCPs, mouse corpus cavernous pericyte; MCECs, mouse cavernous endothelial cells; ns, not significant

**Fig. 3:**
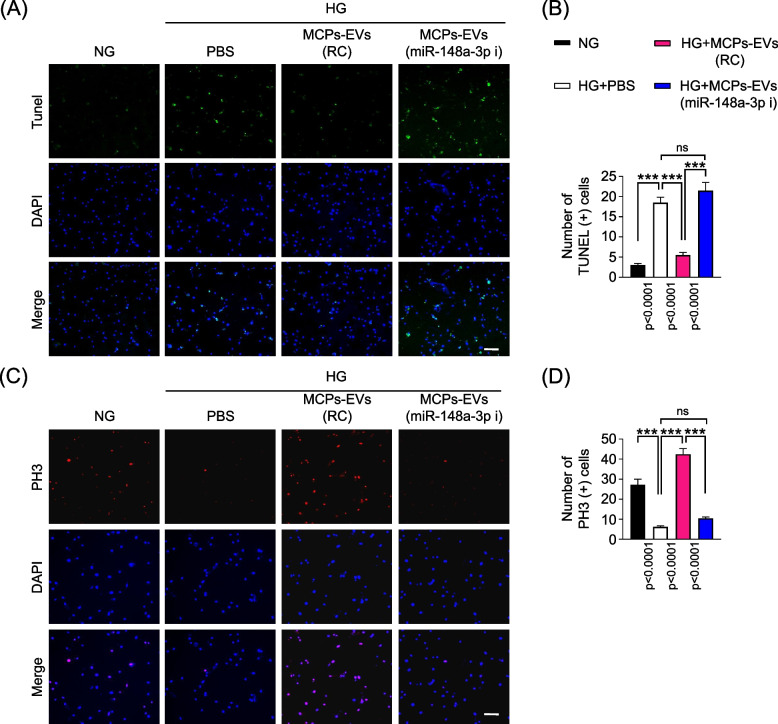
MCPs-EVs decreased apoptosis and increased proliferation of MCECs through miR-148a-3p under high-glucose (HG) conditions. **a** TUNEL (green) immunostaining in MCECs treated with PBS, MCPs-EVs-reagent control (MCPs-EVs-RC, 1 μg/mL), MCPs-EVs-miR-148a-3p inhibitor (MCPs-EVs-miR-148a-3p-i, 1 μg/mL) under normal-glucose (NG) and HG conditions for 3 days. Scale bar = 100 μm. **b** Number of apoptotic cells were quantified by ImageJ and the results are presented as mean ± SEM (n = 4). **c** PH3 (red) immunostaining in MCECs with the same treatment conditions as for TUNEL immunostaining. Nuclear were labeled with DAPI (blue). **d** Number of PH3-positive cells were quantified by ImageJ and the results are presented as mean ± SEM (n = 4). ***p < 0.001. TUNEL, terminal deoxynucleotidyl transferase-mediated deoxyuridine triphosphate nick end labeling; MCPs, mouse corpus cavernous pericyte; MCECs, mouse cavernous endothelial cells; PH3, phospho-Histone H3; ns, not significant; DAPI, 4.6-diamidino-2-phenylindole

To assess the angiogenic effect of miR-148a-3p, which has the highest expression in MCPs-EVs, we transfected a miR-148a-3p inhibitor in MCPs and isolated MCPs-EVs, we found that the expression of miR-148a-3p was significantly reduced in MCPs and MCPs-EVs (Fig. 2a and b). Then, we evaluated the expression of miR-148a-3p and nitrite levels in MCECs, which was treated with PBS, MCPs-EVs-regent control (MCPs-EVs-RC), MCPs-EVs-miR-148a-3p inhibitor (MCPs-EVs-miR-148a-3p-i) under normal-glucose (NG) and high-glucose (HG) conditions. We found that the expression of miR-148a-3p (Fig. 2c) and nitrite levels (Fig. 2d) were significant reduced in MCECs under HG conditions, and MCPs-EVs treatment leads to the recovery of miR-148a-3p expression and nitrite levels. However, there was no significant change in the expression of miR-148a-3p and nitrite levels in the group treated with miR-148a-3p-depleted MCP-EVs under HG conditions. Next, tube-formation (Fig. 2e) and cell-migration (Fig. 2f) abilities were significantly reduced in MCECs exposed to the high-glucose PBS environment. Reagent-controlled MCPs-EVs can induce tube formation and migration of endothelial cells under high-glucose conditions, thereby promoting angiogenesis. However, these effects were significantly attenuated in the group treated with miR-148a-3p-depleted MCPs-EVs under high-glucose conditions (Fig. 2e–h). Furthermore, using TUNEL assay and PH3 staining, we found that miR-148a-3p-depleted MCPs-EVs were unable to reduce apoptosis (Fig. 3)a-b or induce proliferation (Fig. 3c and d) of MCECs under high-glucose conditions. Collectively, these results suggest that miR-148a-3p plays an important role in MCPs-EV-promoted angiogenesis by inducing MCEC migration, survival, and proliferation under high-glucose conditions.

### MCPs-EVs improve erectile function through miR-148a-3p in diabetic mice

**Fig. 4:**
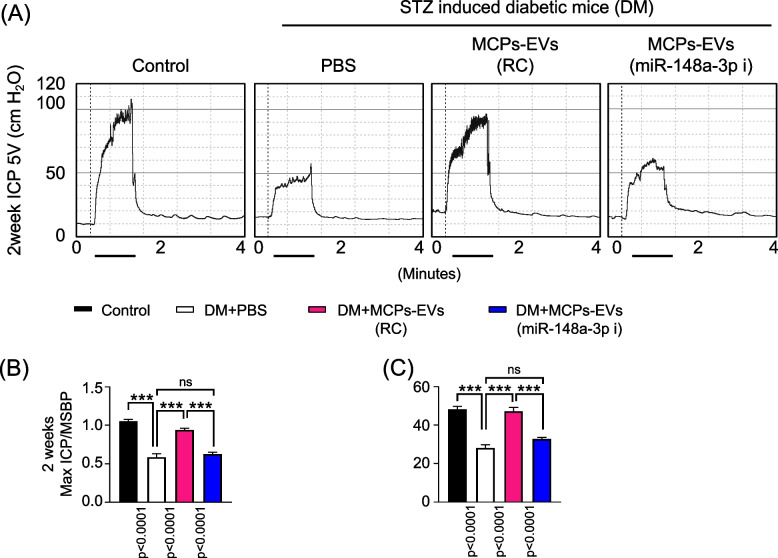
MCPs-EVs improved erectile function through miR-148a-3p in STZ-induced diabetic mice. **a** Representative ICP responses for age-matched nondiabetic controls, diabetic mice stimulated at 2 weeks after intracavernous PBS, MCPs-EVs-reagent control (MCPs-EVs-RC, 5 μg/20 μL), and MCPs-EVs-miR-148a-3p inhibitor (MCPs-EVs-miR-148a-3p-i, 5 μg/20 μL) injection. The cavernous nerve was stimulated at 5 V, and stimulus time is indicated by a solid bar. **b, c** Ratios of mean maximal ICP and total ICP (area under the curve) versus MSBP were calculated for each group. Data in graphs are mean ± SEM (*n* = 5). ***p < 0.001. STZ, streptozotocin; ICP, intracavernous pressure; MSBP, mean systolic blood pressure; MCPs, mouse corpus cavernous pericytes; ns, not significant

**Fig. 5:**
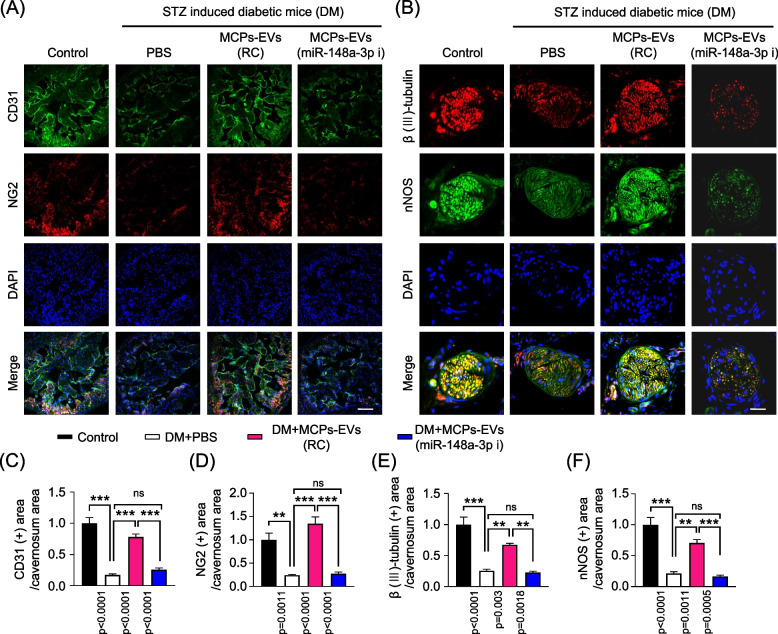
MCPs-EVs improve cavernous endothelial cell, pericytes, and neuronal cell content through miR-148a-3p in STZ-induced diabetic mice. **a** CD31 (green) and NG2 (red) immunostaining in cavernous tissue from age-matched nondiabetic controls, diabetic mice stimulated at 2 weeks after intracavernous PBS, MCPs-EVs-reagent control (MCPs-EVs-RC, 5 μg/20 μL), or MCPs-EVs-miR-148a-3p inhibitor (MCPs-EVs-miR-148a-3p-i, 5 μg/20 μL) injection; scale bar = 100 μm. **b** β (III)-tubulin (red) and nNOS (green) immunostaining in the same cavernous tissue section with the abovementioned CD31 staining groups; scale bar = 25 μm. Nuclear were labeled with DAPI (blue). **c-f** Quantitative analysis of cavernous endothelial cell, pericytes, and β (III)-tubulin- or nNOS-expressing neuronal cell contents using Image J. Data in graphs are presented as mean ± SEM (*n* = 4). The value expressed as ratios of the control group was set to 1. **p < 0.01; ***p < 0.001. STZ, streptozotocin; MCPs, mouse corpus cavernous pericytes; DAPI = 4,6-diamidino-2-phenylindole; ns, not significant

To investigate whether MCPs-EVs have beneficial effects via miR-148a-3p on erectile function in diabetic mice, we intracavernously injected the reagent-controlled or miR-148a-3p-depleted MCPs-EVs and evaluated erectile function 2 weeks later. During electrical stimulation, the ratios of maximal and total intracavernous pressure (ICP) to MSBP were significantly lower in PBS-treated diabetic mice than in age-matched non-diabetic controls. Interestingly, diabetic mice treated with MCPs-EVs had significantly improved erection parameters, reaching almost 93% of the values in controls. However, miR-148a-3p-depleted MCPs-EV treatment showed no such effects in diabetic mice (Fig. 4). Immunofluorescence staining for CD31 (Fig. 5a, c), NG2 (Fig. 5 a, d) in cavernosum tissues and β (III)-tubulin (Fig. 5 b, e) and neuronal NOS (nNOS; Fig. 5 b, f) in dorsal nerve bundles demonstrated that MCPs-EVs significantly improved the endothelial cell, pericyte, and nerve composition in diabetic mice. Fasting and postprandial blood glucose concentrations were significantly higher in diabetic mice than in control mice. However, there were no significant differences in body weight and blood glucose levels in diabetic mice regardless of treatment (Table 2). No detectable differences in MSBP were observed between the three STZ-induced experimental groups. These results suggest that miR-148a-3p plays an important role in MCPs-EVs-induced improvement in erectile function by rescuing cavernous neurovascular regeneration in diabetic mice.

**Table 2:** Physiologic and metabolic parameters: 2 weeks after treatment with PBS, MCPs-EVs (RC), MCPs-EVs (miR-148a-3p i)

		STZ-induced diabetic mice		
	Control	PBS	MCPs-EVs (RC)	MCPs-EVs (miR-148a-3p i)
**Body weight (g)**	31.5 ± 0.6	23.7 ± 1.0*	25.3 ± 0.3*	25.8 ± 0.5*
**Fasting glucose (mg/dl)**	103.6 ± 2.6	551.2 ± 19.8*	567.2 ± 19.3*	549.4 ± 18.2*
**Postprandial glucose (mg/dl)**	168.8 ± 18.0	590.0 ± 6.4*	595.2 ± 4.8*	594.0 ± 5.7*
**MSBP (mm Hg)**	102.0 ± 2.6	101.2 ± 2.7	100.6 ± 1.9	102.0 ± 1.2

Values are the mean ± SEM for *n* = 5 animals per group. *RC* regent control; *miR*-148a-3p i miR-148a-3p inhibitor; *STZ* streptozotocin; *MSBP* Mean systolic blood pressure; *P < 0.05 vs. Control group

### *PDK4* as a target gene of miR-148a-3p

**Fig. 6:**
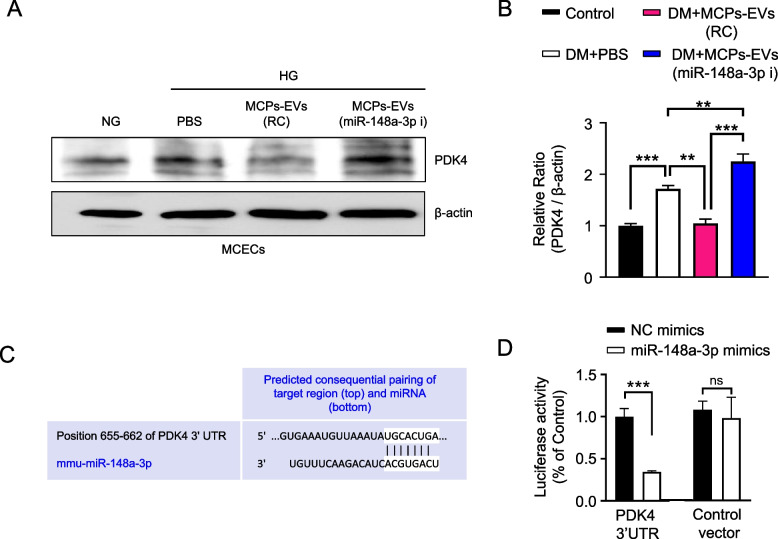
*PDK4* was identified as a target gene of miR-148a-3p. **a** Representative Western blots for *PDK4* in MCECs treated with PBS, MCPs-EVs-reagent control (MCPs-EVs-RC, 1 μg/mL) and MCPs-EVs-miR-148a-3p inhibitor (MCPs-EVs-miR-148a-3p-i, 1 μg/mL) under normal-glucose (NG) and high-glucose (HG) conditions for 3 days. **b** Relative intensities of PDK4 and β-actin on Image J analysis. Data in graphs are presented as mean ± SEM (*n* = 4). Values expressed as ratios of the control group were set to 1. **p < 0.01; ***p < 0.001. **c** The binding sequences of miR-148a-3p on position 655-662 of PDK4 3’UTR. **d** Luciferase reporter assay was used to assess the binding capacity between miR-148a-3p and PDK4 in MCECs. Data in graphs are presented as mean ± SEM (n = 4). Values expressed as ratios of the NC mimics co-transfected with PDK4 3’UTR plasmid group was set to 1. ***p < 0.001. MCPs, mouse corpus cavernous pericyte; MCECs, mouse cavernous endothelial cells; NC mimics, negative control mimics; ns, not significant

There were 98 target genes for miR-148a-3p that were predicted from the DIANA-microT-CDS, TargetScan, and miRDB according to the following parameters: target score > 80, intersection in all three algorithms, and are detected in MCECs through the published RNA-sequencing result [20]. From the literature review and removal of genes to identify a target of miR-148a-3p, we selected 5 predicted target genes: *Ago2, S1pr1, PDK4, Prkaa1*, and *Adam10*. Of these genes, we focused on *PDK4* expression which was significantly regulated by miR-148a-3p (Fig. 6a, b). Using TargetScan, the binding sequences of miR-148a-3p at position 655-662 of PDK4 3’UTR is shown in Fig. 6c. The luciferase reporter assay demonstrated that luciferase activity was significantly reduced in the PDK4 3’UTR plasmid and miR148a-3p-mimic co-transfected group. However, the luciferase activity in the control vector-transfected group showed no difference (Fig. 6d). These results suggest that miR-148a-3p inhibits PDK4 expression by directly binding to the 3’UTR of PDK4 mRNA.

## Discussion

Increasingly, evidence has suggested that miRNAs are widely involved in physiological and pathological processes, including in cancer, diabetes mellitus, and neurological disorders [12, 24, 25]. However, the detailed role of miRNAs in pericyte-derived EVs is unknown. Using small-RNA sequencing analysis, we found that miR-148a-3p had the highest expression in MCPs-EVs. High-glucose conditions promote pericyte dysfunction [21] and reduce miR-148a-3p levels in human retinal microvascular endothelial cells [26]. Furthermore, miR-148a-3p played different roles in angiogenesis due to different microenvironments [27]. Therefore, we investigated whether high-glucose-induced pericyte dysfunction could reduce the secretion of miR-148a-3p-containing MCPs-EVs, thereby reducing angiogenesis under high-glucose conditions. To further investigate the role of miR-148a-3p in the promotion of angiogenesis by MCPs-EVs, we depleted the miR-148a-3p in MCPs-EVs and found that the angiogenic effect was significantly reduced under high-glucose conditions. Thus, miR-148a-3p may play an important role in MCPs-EV-induced angiogenesis.

Next, we used a diabetes-induced ED mouse model to evaluate the neurovascular regenerative effects of MCPs-EVs as described previously [21, 22]. We found that MCPs-EVs significantly improved erectile function by inducing endothelial cell, pericyte, and neuronal cell content upregulation in the cavernosal tissues of diabetic mice. However, miR-148a-3p-depleted MCPs-EVs did not improve erectile function in diabetic mice. Therefore, MCPs-EVs can function as an intercellular delivery tool for miR-148a-3p transfer into recipient cells for improving erectile function in diabetic mice.

To demonstrate how miR-148a-3p regulates erectile function in patients with diabetes, we reviewed the literature related to miR-148a-3p found in endothelial cells (HUVECs) (PMID: 31723119). Multiple targets have been identified, such as NRP-1, ROBO1, and ITGa5. But in order to find targets in MCECs, we used three miRNA target prediction programs and subsequent verification experiments, and we found that PDK4 is a regulated target by miR-148a-3p. The luciferase assay results demonstrated that miR-148a-3p directly targets the 3’UTR of PDK4, thereby reducing PDK4 expression. The PDK4 level is elevated in patients with diabetes, and a PDK inhibitor enhances insulin activity by promoting glucose oxidation [28]. In addition, reducing PDK4 expression with siRNAs in pulmonary arterial hypertension-induced pericytes can enhance the endothelial–pericyte interaction [29]. Thus, we hypothesized that miR-148a-3p transferred by MCPs-EVs might reduce PDK4 expression, thereby promoting neurovascular regeneration in diabetic mice. However, this study did not confirm whether decreased PDK4 expression in the MCPs-EVs treatment group directly affected the erection in diabetic ED mice. Nonetheless, this study provides a basis for understanding the detailed mechanisms and therapeutic value of MCPs-EVs in improving erection in diabetes-induced ED.

In this study, we showed, for the first time, that MCPs-EVs improve erectile function in diabetic mice in a miR-148a-3p-dependent manner. However, our study has some limitations. First, we did not verify whether the known targets regulated by miR-148a-3p (NRP-1, ROBO1, and ITGa5) in endothelial cells (HUVEC) also apply to MCECs. In the future, studies of known targets of miR-148a-3p may be help us to further understood the specific mechanism of miR-148a-3p in diabetic ED. Second, we could not evaluate the expression of miR-148a-3p and nitrite levels in in vivo study. Third, we could not evaluate whether repression of PDK4 expression by miR-148a-3p had a direct effect on erection in diabetes-induced ED. Studies are needed to evaluate the detailed mechanism and function of PDK4 in ED and other vascular and/ or neurological disorders.

## Conclusions

Our study shows that miR-148a-3p, which is highly expressed in MCPs-EVs, plays an important role in enhancing neurovascular regeneration and ultimately improving erectile function by inhibiting PDK4 expression in diabetic mice.

### Supplementary Information

**Additional file 1.**

## Data Availability

They are available from the corresponding author on special request.

## Abbreviations

MCP: Mouse corpus cavernous pericyte
EVs: Extracellular vesicles
ED: Erectile dysfunction
MCECs: Mouse cavernous endothelial cell
PDK4: Pyruvate dehydrogenase kinase-4
PDE5: Phosphodiesterase 5
NO: Nitric oxide
DM: Diabetes mellitus
mRNAs: Messenger RNAs
miRNAs: MicroRNAs
NV: Nanovesicles
CNI: Cavernous nerve injury
TUNEL: Terminal deoxynucleotidyl transferase-mediated deoxyuridine triphosphate nick-end labeling
DAPI: 4,6-diamidino-2-phenylindole
ICP: Intracavernous pressure
MSBP: Mean systolic blood pressure
PH3: Phospho-Histone H3
FITC: Fluorescein isothiocyanate
TRITC: Tetramethylrhodamine
NG: Normal-glucose
HG: High-glucose
STZ: Streptozotocin

## References

[1] Ayta IA, McKinlay JB, Krane RJ (1999). The likely worldwide increase in erectile dysfunction between 1995 and 2025 and some possible policy consequences. BJU Int.

[2] Huang SA, Lie JD (2013). Phosphodiesterase-5 (PDE5) inhibitors in the Management of Erectile Dysfunction. P T.

[3] Giri B, Dey S, Das T, Sarkar M, Banerjee J, Dash SK (2018). Chronic hyperglycemia mediated physiological alteration and metabolic distortion leads to organ dysfunction, infection, cancer progression and other pathophysiological consequences: an update on glucose toxicity. Biomed Pharmacother.

[4] Kolluru GK, Bir SC, Kevil CG (2012). Endothelial dysfunction and diabetes: effects on angiogenesis, vascular remodeling, and wound healing. Int J Vasc Med.

[5] Jin HR, Kim WJ, Song JS, Piao S, Choi MJ, Tumurbaatar M, Shin SH, Yin GN, Koh GY, Ryu JK, Suh JK (2011). Intracavernous delivery of a designed angiopoietin-1 variant rescues erectile function by enhancing endothelial regeneration in the streptozotocin-induced diabetic mouse. Diabetes.

[6] Burchardt M, Burchardt T, Anastasiadis AG, Buttyan R, de la Taille A, Shabsigh A, Frank J, Shabsigh R (2005). Application of angiogenic factors for therapy of erectile dysfunction: protein and DNA transfer of VEGF 165 into the rat penis. Urology.

[7] Hu L, Qi S, Zhang K, Fu Q (2018). Essential role of brain-derived neurotrophic factor (bdnf) in diabetic erectile dysfunction. Andrologia.

[8] Doyle LM, Wang MZ. Overview of extracellular vesicles, their origin, composition, purpose, and methods for exosome isolation and analysis. Cells. 2019;8(7) 10.3390/cells8070727. 10.3390/cells8070727 PMC6678302 31311206

[9] O'Brien K, Breyne K, Ughetto S, Laurent LC, Breakefield XO (2020). RNA delivery by extracellular vesicles in mammalian cells and its applications. Nat Rev Mol Cell Biol.

[10] Kwon MH, Song KM, Limanjaya A, Choi MJ, Ghatak K, Nguyen NM, Ock J, Yin GN, Kang JH, Lee MR, Gho YS, Ryu JK, Suh JK (2019). Embryonic stem cell-derived extracellular vesicle-mimetic nanovesicles rescue erectile function by enhancing penile neurovascular regeneration in the streptozotocin-induced diabetic mouse. Sci Rep.

[11] Yin GN, Park SH, Ock J, Choi MJ, Limanjaya A, Ghatak K, Song KM, Kwon MH, Kim DK, Gho YS, Suh JK, Ryu JK (2020). Pericyte-derived extracellular vesicle-mimetic Nanovesicles restore erectile function by enhancing neurovascular regeneration in a mouse model of cavernous nerve injury. J Sex Med.

[12] Kang J, Song Y, Zhang Z, Wang S, Lu Y, Liu X. Identification of key microRNAs in diabetes mellitus erectile dysfunction rats with stem cell therapy by Bioinformatic analysis of deep sequencing data. World J Mens Health. 2022; 10.5534/wjmh.210147. 10.5534/wjmh.210147 PMC9482859 35021304

[13] Lee JY, Ryu DS, Kim WJ, Kim SJ (2016). Aberrantly expressed microRNAs in the context of bladder tumorigenesis. Investig Clin Urol.

[14] Ock J, Suh JK, Hong SS, Kang JH, Yin GN, Ryu JK (2023). IGFBP5 antisense and short hairpin RNA (shRNA) constructs improve erectile function by inducing cavernosum angiogenesis in diabetic mice. Andrology.

[15] Yin GN, Kim DK, Kang JI, Im Y, Lee DS, Han AR, Ock J, Choi MJ, Kwon MH, Limanjaya A, Jung SB, Yang J, Min KW, Yun J, Koh Y, Park JE, Hwang D, Suh JK, Ryu JK, Kim HM (2022). Latrophilin-2 is a novel receptor of LRG1 that rescues vascular and neurological abnormalities and restores diabetic erectile function. Exp Mol Med.

[16] Yin GN, Das ND, Choi MJ, Song KM, Kwon MH, Ock J, Limanjaya A, Ghatak K, Kim WJ, Hyun JS, Koh GY, Ryu JK, Suh JK (2015). The pericyte as a cellular regulator of penile erection and a novel therapeutic target for erectile dysfunction. Sci Rep.

[17] Yin GN, Ryu JK, Kwon MH, Shin SH, Jin HR, Song KM, Choi MJ, Kang DY, Kim WJ, Suh JK (2012). Matrigel-based sprouting endothelial cell culture system from mouse corpus cavernosum is potentially useful for the study of endothelial and erectile dysfunction related to high-glucose exposure. J Sex Med.

[18] Yin GN, Ock J, Choi MJ, Song KM, Ghatak K, Minh NN, Kwon MH, Seong DH, Jin HR, Ryu JK, Suh JK (2020). A simple and nonenzymatic method to isolate human Corpus Cavernosum endothelial cells and Pericytes for the study of erectile dysfunction. World J Mens Health.

[19] Zhao L, Yu J, Wang J, Li H, Che J, Cao B (2017). Isolation and identification of miRNAs in exosomes derived from serum of colon cancer patients. J Cancer.

[20] Yin GN, Ock J, Choi MJ, Limanjaya A, Ghatak K, Song KM, Kwon MH, Suh JK, Ryu JK (2021). Gene expression profiling of mouse cavernous endothelial cells for diagnostic targets in diabetes-induced erectile dysfunction. Investig Clin Urol.

[21] Yin GN, Wu J, Cui Y, Lin C, Shi L, Gao ZL, Suh JK, Ryu JK, Jin HR (2021). Transcriptional profiling of mouse cavernous pericytes under high-glucose conditions: implications for diabetic angiopathy. Investig Clin Urol.

[22] Jin HR, Kim WJ, Song JS, Choi MJ, Piao S, Shin SH, Tumurbaatar M, Tuvshintur B, Nam MS, Ryu JK, Suh JK (2009). Functional and morphologic characterizations of the diabetic mouse corpus cavernosum: comparison of a multiple low-dose and a single high-dose streptozotocin protocols. J Sex Med.

[23] Anita L, Yin GN, Hong SS, Kang JH, Gho YS, Suh JK, Ryu JK (2022). Pericyte-derived extracellular vesicle-mimetic nanovesicles ameliorate erectile dysfunction via lipocalin 2 in diabetic mice. Int J Biol Sci.

[24] Enokida H, Yoshino H, Matsushita R, Nakagawa M (2016). The role of microRNAs in bladder cancer. Investig Clin Urol.

[25] Kamal MA, Mushtaq G, Greig NH (2015). Current update on synopsis of miRNA dysregulation in neurological disorders. CNS Neurol Disord Drug Targets.

[26] Wang J, Yao Y, Wang K, Li J, Chu T, Shen H (2020). MicroRNA-148a-3p alleviates high glucose-induced diabetic retinopathy by targeting TGFB2 and FGF2. Acta Diabetol.

[27] Cai Q, Zhu A, Gong L (2018). Exosomes of glioma cells deliver miR-148a to promote proliferation and metastasis of glioblastoma via targeting CADM1. Bull Cancer.

[28] Lee IK (2014). The role of pyruvate dehydrogenase kinase in diabetes and obesity. Diabetes Metab J.

[29] Yuan K, Shao NY, Hennigs JK, Discipulo M, Orcholski ME, Shamskhou E, Richter A, Hu X, Wu JC, de Jesus Perez VA (2016). Increased pyruvate dehydrogenase kinase 4 expression in lung Pericytes is associated with reduced endothelial-Pericyte interactions and small vessel loss in pulmonary arterial hypertension. Am J Pathol.

