# Correction: MicroRNA-148a-3p in pericyte-derived extracellular vesicles improves erectile function in diabetic mice by promoting cavernous neurovascular regeneration

**DOI:** 10.1186/s12894-023-01399-z

**Published:** 2024-01-13

**Authors:** Jiyeon Ock, Fang-Yuan Liu, Fitri Rahma Fridayana, Lashkari Niloofar, Minh Nhat Vo, Yan Huang, Shuguang Piao, Tie Zhou, Yin Guonan

**Affiliations:** 1 Department of Urology and National Research Center for Sexual Medicine, Inha University School of Medicine, 7-206, 3rd ST, Shinheung-Dong, Jung-Gu, Incheon, 22332 Republic of Korea; 2 Program in Biomedical Science & Engineering, Inha University, Incheon, South Korea; 3 Department of Urology, Changhai Hospital Affiliated with the Naval Medicine University, Tie Zhou, Shanghai, 200433 People’s Republic of China; 4 Department of Urology, Shanghai Fourth People’s Hospital, School of Medicine, Tongji University, No. 1279 Sanmen Road, Shanghai, 200434 China

**BMC Urology (2023) 23:209**

10.1186/s12894-023-01378-4

The author name “Yin Guonan” was incorrectly written as “Yin Gounan” and the same has been updated.

The original article has been corrected.

