# Supplementary figures and images for: MicroRNA-148a-3p in pericyte-derived extracellular vesicles improves erectile function in diabetic mice by promoting cavernous neurovascular regeneration
Source: BMC Urol. 2023 Dec 16;23:209. doi: 10.1186/s12894-023-01378-4 (PMC10725581; doi:10.1186/s12894-023-01378-4)

Figure 1B related uncutted gel images

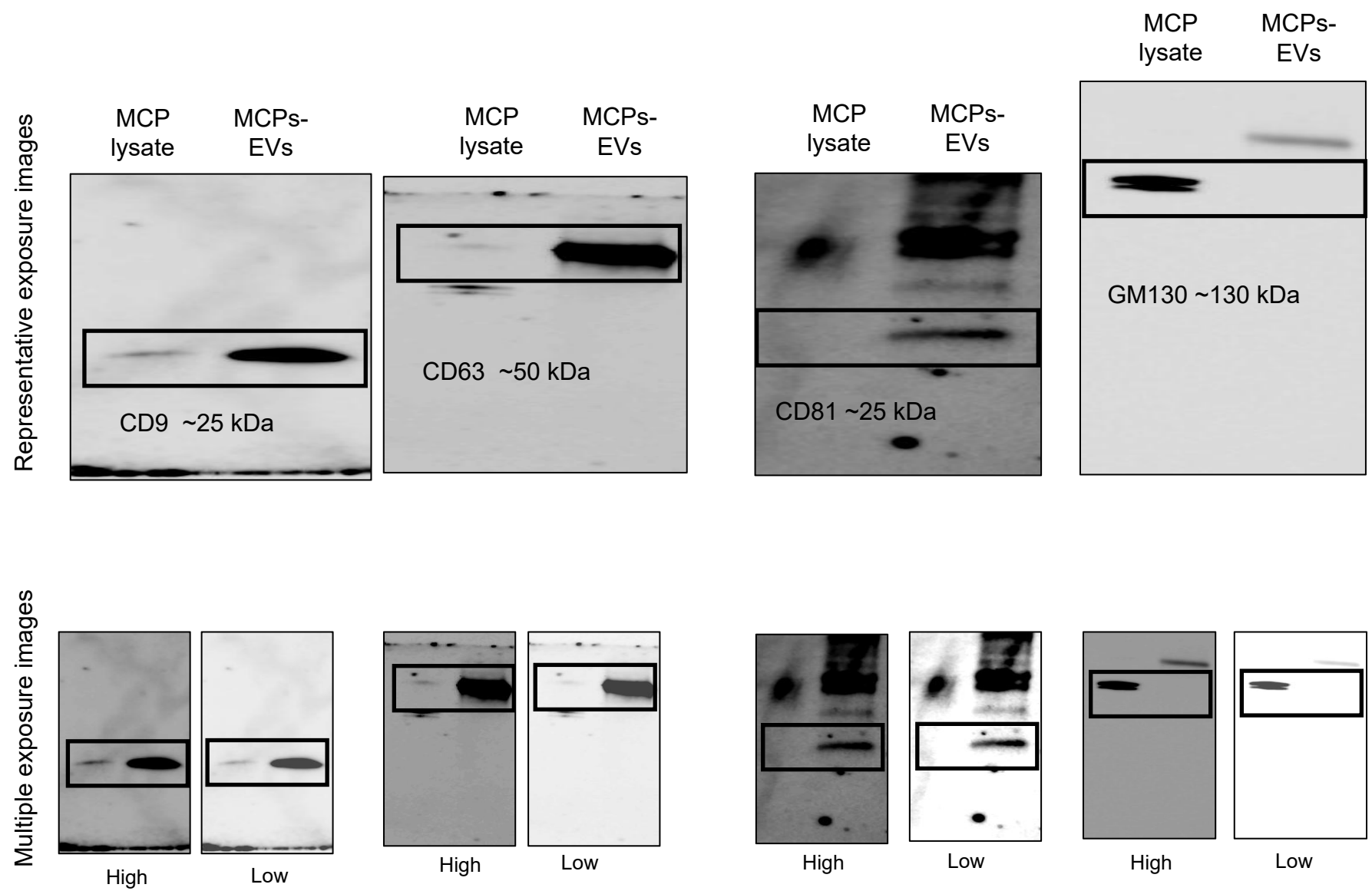

Figure 6A related uncutted gel images

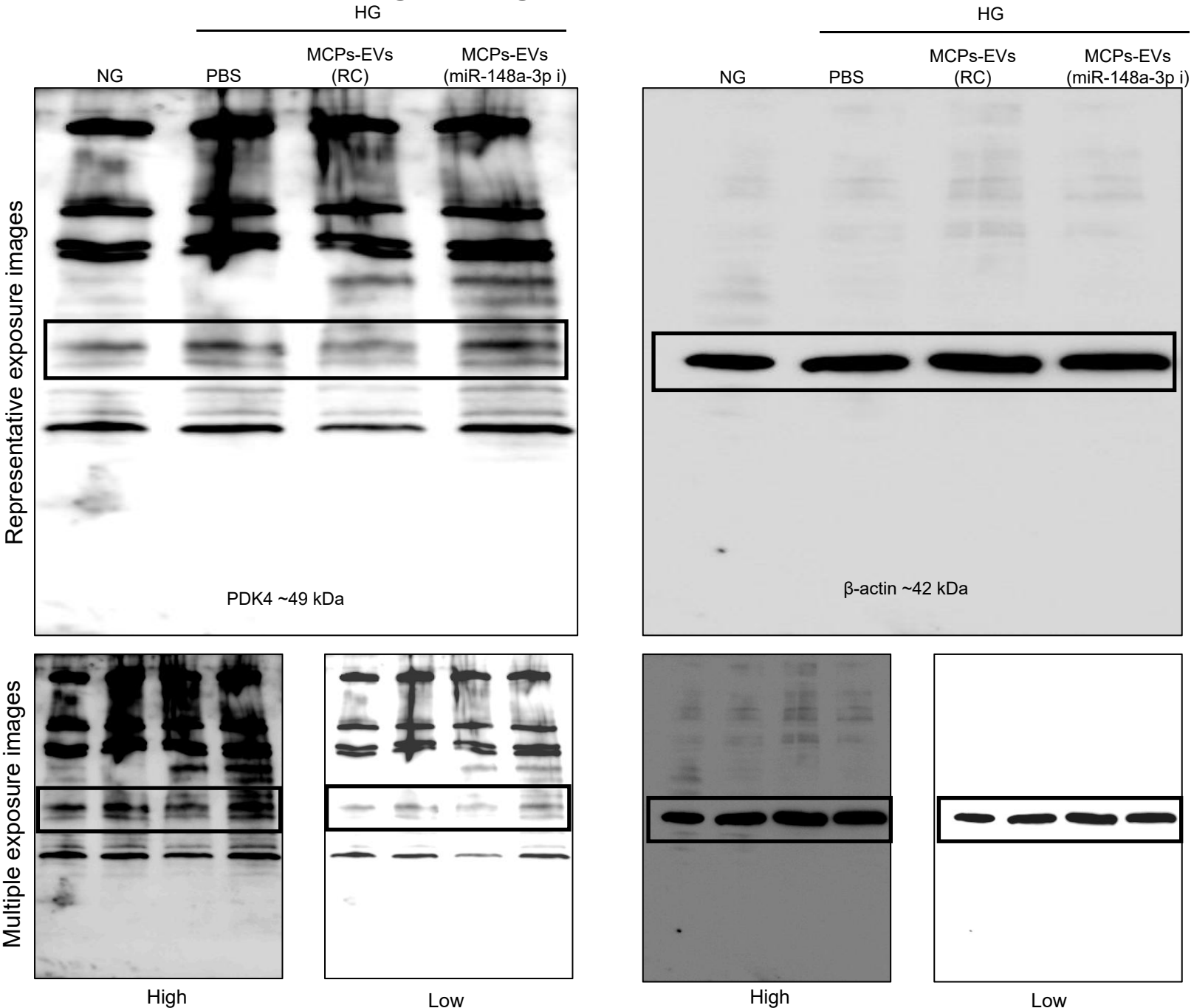

Supplement: Supplementary file 1 — Additional file 1. [file 12894_2023_1378_MOESM1_ESM.pdf]
